# End-of-Life Care of Assisted Living Residents with Advanced Dementia: Views of Informal Caregivers

**DOI:** 10.1093/geroni/igaf122.1696

**Published:** 2025-12-31

**Authors:** Molly Perkins, Michael Lepore, Mi-Kyung Song, Louise Savoye, Shermetria Massingale, Jeffrey Lentz, Nidhi Ramprasad, Alexis Bender

**Affiliations:** Emory University School of Medicine, Atlanta, Georgia, United States; University of Massachusetts Amherst, Amherst, Massachusetts, United States; Emory University, Atlanta, Georgia, United States; Emory University School of Medicine, Atlanta, Georgia, United States; Emory University School of Medicine, Atlanta, Georgia, United States; Emory University School of Medicine, Atlanta, Georgia, United States; Emory University School of Medicine, Atlanta, Georgia, United States; Emory University, Atlanta, Georgia, United States

## Abstract

Most persons with dementia in the U.S. die in long-term care communities. Assisted living (AL), the fastest growing long-term care option in the U.S., is a primary provider of this end-of-life (EOL) care. To date, little is known regarding the caregiving experiences of informal caregivers (relatives and friends) who are providing care for the growing population of AL residents with advanced dementia at end of life. Using data from interviews with 25 informal caregivers of recently deceased AL residents with advanced dementia enrolled in a 5-year NIA-funded (RF1AG069114/R01AG069114) longitudinal project, this study begins to address this important knowledge gap. Semi-structured interviews were digitally recorded, transcribed verbatim, and analyzed using thematic analysis. Most participants (mean age = 64) were female (88%), Non-Hispanic White (88%), and had some college education or higher (84%). Most residents (96%) were on hospice at the time of their death. Some participants described the “teamwork” they observed among AL staff and hospice workers as important to the quality of EOL care residents received. Others described a lack of coordination and collaboration, including some conflict, among these care providers as their biggest challenge. A need for more communication between informal caregivers, AL staff, and hospice regarding everyone’s role during EOL was noted by some, including concerns about inadequate pain management and insufficient monitoring of vital signs, and other care needs. Findings have important implications for interventions to improve the EOL experience of AL residents with advanced dementia and their care partners.

